# Evaluation of the efficacy of transient overvoltages suppression measures in different wind farm topologies using SF6 circuit breaker

**DOI:** 10.1038/s41598-023-40768-4

**Published:** 2023-08-22

**Authors:** Tamer Eliyan, Fady Wadie

**Affiliations:** 1 Faculty of Engineering at Shoubra, Benha University, Cairo, 11629 Egypt; 2 Department of Electrical Power & Machines Engineering Department, The Higher Institute of Engineering at El-Shorouk City, Alshorouk Academy, Cairo, 11837 Egypt; 3 Mechatronics and Robotics Engineering Department, Faculty of Engineering, Egyptian Russian University, Badr City, Egypt

**Keywords:** Electrical and electronic engineering, Power distribution, Power stations

## Abstract

Various overvoltage mitigation schemes were used in literature in suppression of switching overvoltages in wind farms. However, the evaluation of how the effectiveness of these mitigation techniques would vary with the change of the wind farm topology is still un-explored territory. The main aim of this paper is to study the effectiveness of four mitigation schemes while using SF6 circuit breaker namely; R–L smart choke, R–C snubber circuit, surge capacitor and pre-insertion resistor (PIR) were studied in four different wind farm topologies; radial, single-sided ring, double-sided ring and star topologies. The topologies were based on a real wind farm located in Zaafrana, Egypt. The results showed that R–L choke to be the most effective scheme for all topologies followed by PIR, R–C snubber and surge capacitor schemes respectively. Their percentage of reduction of overvoltage ranged from 62 to 84% for R–L choke, 33–67% for PIR, 8–25% for R–C snubber circuits and 4–15% for surge capacitors. Also, it was shown that the change of the wind farm topology didn’t affect the order of effectiveness of the mitigation schemes such that R–L remained the most effective and surge capacitor the least effective for all topologies.

## Introduction

The dual sided problem arising from the vast scale of the energy market due to the increasing demand for electric power in addition to the diminishing supply for the reserve fossil fuel has led to a fast track reliance on renewable energy sources. One of the main sources that has been utilized globally is wind energy leading to excessive investigation into the performance and protection wind farms. However, the structure of wind farms differs from conventional power stations in employing huge numbers of power transformers, underground cables that extend for long distances and control algorithms that mandate frequent switching operations^1^. Generally, wind farms constitute of several electrical and mechanical elements such as wind towers, turbines, underground cables, transformers, and protection devices. The connections between these elements could be built in various topologies with four main widely known topologies namely; topologies are Radial topology, Single-Sided Ring (SSR) topology, Double-Sided Ring (DSR) topology, and Star topology^2^.

The frequent switching induces a transient overvoltage whose destructive effect is amplified by the presence of power transformers and the MV cables which forms a resonant RLC circuit^3^. This destructive effect has led to insulation failures in wind farms^4^. The consequent losses due to theses failures have lead researchers to investigate the impact of transient overvoltages in wind farms^5–7^. The highlights of the literature regarding the study of the impact of overvoltages in wind farms and the suppression measures employed to mitigate the overvoltages are summarized in Table 1. The main focus of the survey was upon recent papers within the last five years. For such reason, the table scans most papers within years 2019 up to 2023 with a total of 18 publications in the recent five years and 3 publications in earlier years.

**Table 1:** Highlights in literature investigating the impact of overvoltages in wind farms.

References	Year	Topic studied in publication	Suppression measures used
^3^	2021	Investigated switching overvoltages in different wind farm topologies	Splitting the switching process into two stages
^8^	2022	Proposed a method for calculating arcing time and probability of reignitions	RC Snubber circuit and phase controlled switching
^9,11^	2021, 2022	Proposed a method that evaluates the of suppression measures of overvoltages	Surge arrester, RC snubber
^10^	2019	Analyzed the factors affecting switching transient overvoltage in an offshore wind farm	Not discussed
^12^	2023	Investigated the arising temporary overvoltage resulting from de-energization conditions, and the Ferro resonance in off shore wind farms	Pre-Insertion Resistor (PIR)
^13^	2020	Analyzed statistically the overvoltage variations across the step-up transformer during the switching of circuit breaker	Not discussed
^14^	2021	Investigated the overvoltage caused by switching off shunt reactors in a 35 kV substation,	Phase-controlled VCB, surge arresters, RC snubber, double-break circuit breaker and increasing the cable length
^15^	2020	Investigated temporary overvoltage arising from switching of the circuit breaker connecting the wind turbine to the feeder	A damping resistor, shunt reactor and surge arrester
^16^	2019	Performed sensitivity analysis to define the factors affecting transient overvoltages in off-shore wind farms	Not discussed
^17^	2019	Analyzed the over-voltage formation mechanism in HVDC connected wind farm integration system	Proposed control strategies to suppress the over-voltage
^18^	2019	Studied the influence of different grinding parameter upon transient overvoltages	Not discussed
^19,20^	2014, 2011	Switching over-voltages have been simulated in a wind farm to show the effect of changing the topology from radial to star topology in	Not discussed
^21^	2012	Studied the transient overvoltage in a radial system topology of a real practical wind farm	Not discussed
^22^	2022	Studied the impact of multiple reignitions during switching off of vacuum circuit breaker in an offshore wind farm	R–C snubber circuit
^23^	2020	Investigated the impact of switching reignition overvoltage in vacuum circuit breakers when switching off inductive loads	R–C snubber circuit
^24^	2023	Investigated impact of the parameters of vacuum circuit breaker upon the switching overvoltages	Not discussed
^25^	2021	Investigated switching transients in off-shore wind farms	R–C snubber circuit
^26^	2023	Studied the switching overvoltages generated by the breaking of the vacuum circuit breaker in the wind farm	R–C snubber circuit, Choke coil
^27^	2019	Studied the transient overvoltages in off-shore wind farms	Not discussed

The table shows the diverse topics studied in literature in regards of the transient analysis within wind farms. However, the impact of selecting the most suitable suppression technique with respect to the wind farm topology was not investigated before. Such topic is highly important as the degree of severity of the switching overvoltages (SOV) depends mainly on the wind farm topology^3,19,20^. Therefore, the main problem that this paper aims to address is to investigate the most suitable suppression measure for each wind farm topology. Thus, the contributions of this paper will be:

1. Studying and investigating the impact of different wind farm topologies (radial, DSR, SSR and star) upon the transient SOV. Such comparison was rarely covered in literature with few publications as reference 3 only covering this point.
2. Performing simulations to reach for the most suitable suppression technique for each wind farm topology with that particular contribution not investigated before in literature.
3. Presenting conclusions based on the presented results which can be used by researchers for selection the optimal suppression measures based upon network topology. The results showed the R–L choke coil to be the most effective suppression measure in reducing SOV with a percentage of reduction in amplitude of SOV of 62–84%.

The rest of this paper is organized as follows. In “System under study” section presents the system under study. In “Modeling of the system” section discusses the modeling methodologies used for different elements within the system. Simulation results are presented for each wind farm topology in “Simulation results” section. The results of “Discussion and effectivity analysis” section are discussed in “Discussion and effectivity analysis” section showing the main features for selecting the suitable suppression method for each topology. Finally, conclusions are drawn in Sect.  Conclusions.

## System under study

**Figure 1:**
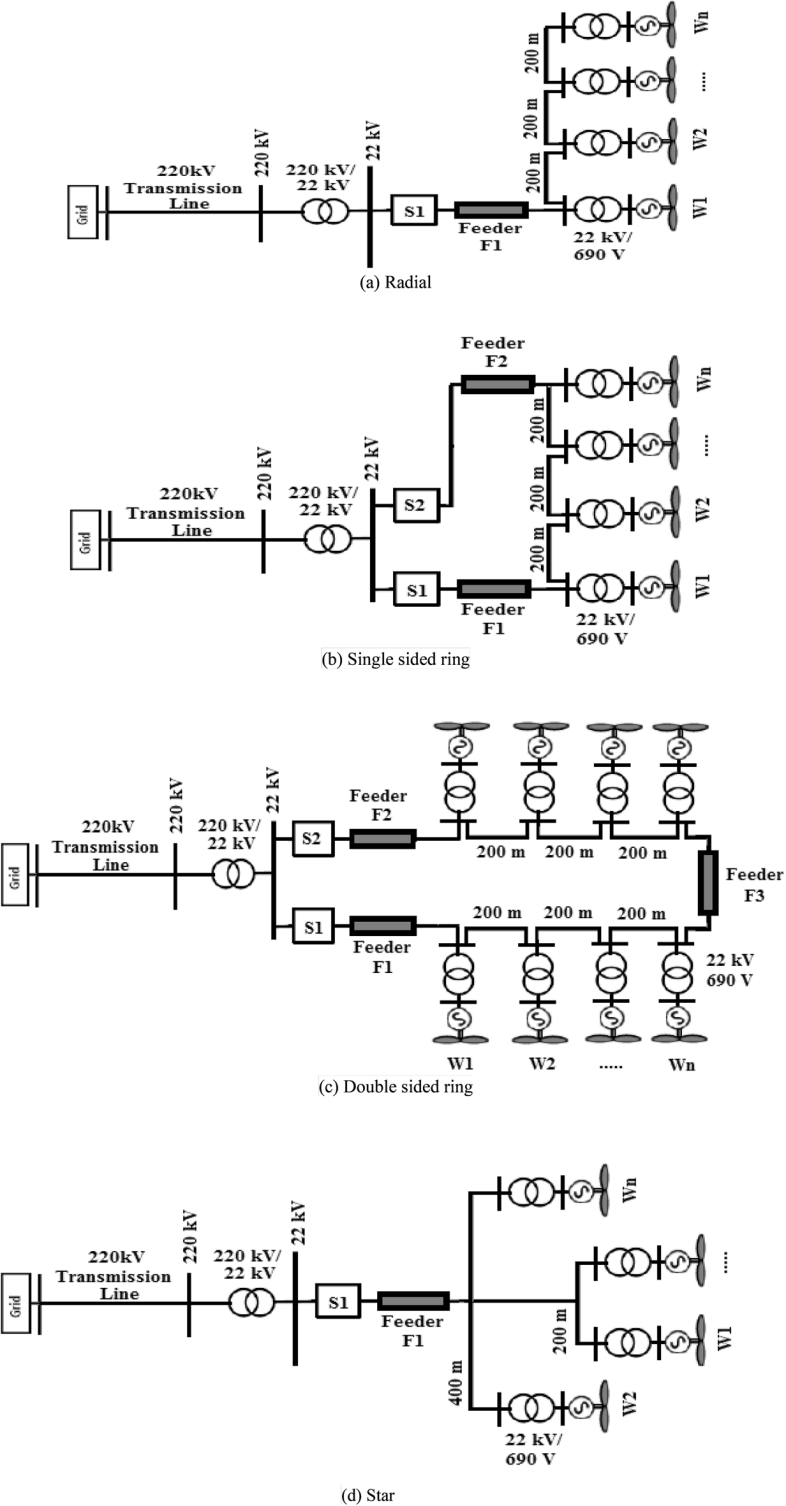
Topologies of wind farm.

The system selected to be under study is based on a real system located in Zaafrana, Egypt. The system is rated at 550 MW generated from 700 wind turbines that were assumed to have identical characteristics. For each turbine, a 1 MVA 690 V/22 kV-transformer is used. Cables of 200-m length are used to connect each two consecutive series turbines. The wind farm is connected to the grid through a 220/22 kV substation. The basic configuration will be kept unchanged but the connection between the turbines will changed to include four different connection topologies namely; radial, single-sided ring, double-sided ring and star connection that are shown in Fig. 1. The lengths of feeders are as follows: feeder F1 is 8 km for all topologies, feeder F2 is 10.4 km in single-sided ring topology and 6.5 km in double-sided ring topology. The length of feeder F3 is 1 km. the length of the cables between series turbines is 200 m for all topologies except of the star topology. In that particular topology, the length of the cable for each turbine will be 200 m for W1, 400 m for W2, 200 m W3, and 400 m for W4. The modifications done is based upon those presented in^3^. The modeling of each component of the system is discussed in the next section.

## Modeling of the system

### Modeling of the circuit breaker

The circuit breakers used in this study are SF6 circuit breakers which could be modeled using several approaches with two most famous modeling blocks used known as Mayr’s and Cassie models. Both models are discussed in the next subsections.

### Mayr’s model

The approach used in Mayr’s model depends upon using dynamic analysis for the arc which defines the breakers ability to reach successful opening^28^. The main variable computed with the arc analysis is the conductance of the arc. During the opening process, the arc goes through four stages that represent the transition process of the breaker from certain state to another. These stages are closed breaker, arcing, arc extinguishing and open stages^29,30^. For the first stage that represents closed breaker and last stage that represents open breaker, the breaker is represented as a constant resistor of negligibly small value of 1 μΩ and high value of MΩ respectively. For the remaining transition stages, a series connection could be deduced as follows^29,31^.

- During the arcing stage, an imbalance arises in between the heating power from the arc ${(\mathrm{P}}_{\mathrm{H}})$ and the cooling power due to the dissipation of the energy from the arc ${(\mathrm{P}}_{\mathrm{o}})$. The difference between both energies is stored within the arc column Q(t) as given in (1)
  1 $$\frac{\mathrm{dQ}(\mathrm{t})}{\mathrm{dt}}={\mathrm{P}}_{\mathrm{H}}- {\mathrm{P}}_{\mathrm{o}}$$
- The energy stored in arc Q(t) is used to define the conductance ${\mathrm{g}}_{\mathrm{m}}\left(\mathrm{t}\right)$ as given in (2) where τ is the arc time constant.
  2 $${\mathrm{g}}_{\mathrm{m}}\left(\mathrm{t}\right)=\mathrm{K }\frac{\mathrm{Q}(\mathrm{t})}{{\mathrm{P}}_{\mathrm{o}}\uptau }$$
- The given expression in (1) could re-defined in terms of the arc conductance as given in (3).
  3 $$\frac{\mathrm{dQ}(\mathrm{t})}{{\mathrm{dg}}_{\mathrm{m}}}\frac{{\mathrm{dg}}_{\mathrm{m}}}{\mathrm{dt}}={\mathrm{P}}_{\mathrm{H}}- {\mathrm{P}}_{\mathrm{o}}$$
- (2) could be substituted in (3) while considering the heating power equal to the amount of electrical power from the arc (v $\times$ i), where v is the arc voltage and i is the arc current resulting in Eq. (4)
  4 $$\frac{{\mathrm{P}}_{\mathrm{o}}\uptau }{{\mathrm{g}}_{\mathrm{m}}} \frac{{\mathrm{dg}}_{\mathrm{m}}}{\mathrm{dt}}=(\mathrm{v}\times \mathrm{i})- {\mathrm{P}}_{\mathrm{o}}$$
- Finally, the conductance ${\mathrm{g}}_{\mathrm{m}}=\mathrm{v}/\mathrm{i}$ could be used in (4) to get (5).
  5 $$\frac{{\mathrm{dg}}_{\mathrm{m}}}{\mathrm{dt}}=\frac{1}{\tau }\left(\frac{{\mathrm{i}}^{2}}{ {\mathrm{P}}_{\mathrm{o}}}- {\mathrm{g}}_{\mathrm{m}}\right)$$

The modeling of the SF6 interrupter into ATP/EMTP environment is done by the MODELS component. That component allows the user to build a coded program integrates its programming with the simulated electrical system.

### Transmission lines, feeders and cables

The modeling of the transmission lines was done by the frequency-dependent model of the transmission lines with their parameters as given in Table 2. While for cables and feeders, the frequency-dependent cable model used with their lengths as given in previous section.

**Table 2:** Transmission line parameters.

	Positive and negative sequence parameters	Zero sequence parameters
Resistance (Ω/km)	0.03	0.13
Reactance (Ω/km)	0.306	0.83
Susceptance (mS/km)	3.25	2.3

### Power transformers

The frequency-dependent transformer model is used to account for the nonlinearities when studying the transformer’s energization. For transient analysis, the effect of the stray capacitance is crucial to be considered. For such reason, the stray capacitances between each winding and the ground and the capacitance between the two windings of the main transformers were simulated using capacitive elements connected across transformer component upon ATP^3,32^.

### Wind turbine

The generation system within the wind turbine consists of different devices including the generator, gearbox, rectifier, three-phase inverter, and other components. Since the main focus of this research is the response from the switching circuit breaker, a 5 MW, 690 V synchronous generator model is used. The leakage reactance of the generator is 0.1 H^32^.

## Simulation results

The investigation of the effectiveness of different mitigation techniques with respect to the topology of the wind farm was undergone using ATP/EMTP simulation platform. Different wind farm topologies provided earlier and shown in Fig. 1 were modeled as described in modeling section. For each topology, different mitigation techniques were applied individually one by one to assess their capability in reducing the switching overvoltages. The mitigation techniques studied include four schemes that showed their effectiveness in literature namely; RC snubber circuit^8,9,11^, Pre-Insertion Resistor (PIR)^12^ and surge capacitor^33^. The value of the parameters of each scheme (R and/or C) were set to reach maximum reduction in overvoltages based on defined ranges from literature^34^. The selected values were 100 Ω for the PIR, R = 50 and C = 1 µF for the RC snubber circuit and the capacitance of the surge capacitor was 1 µF. A fourth technique termed as smart choke is used which was introduced by ABB, which consists of a set of parallel RL filters series connected at the upstream of a protected transformer^35^. The R–L are integrated in their effect with the phase-to-ground capacitance of the transformer forming a low-pass filter that helps in reducing du/dt, limiting the overvoltage levels. The range of different parameters of the R–L is defined by ranges of 25–50 Ω for the damping resistor and 0.6–1.5 mH for inductor^36^. For this work, the previous range of the values were tested and the values giving maximum reduction in switching overvoltage were selected which was found to be R of 50 Ω and inductance of 1.5 mH. The results for different mitigation techniques for each topology with R, L and C values were set as defined earlier are present in the following subsections.

### Simulation results for radial topology

**Figure 2:**
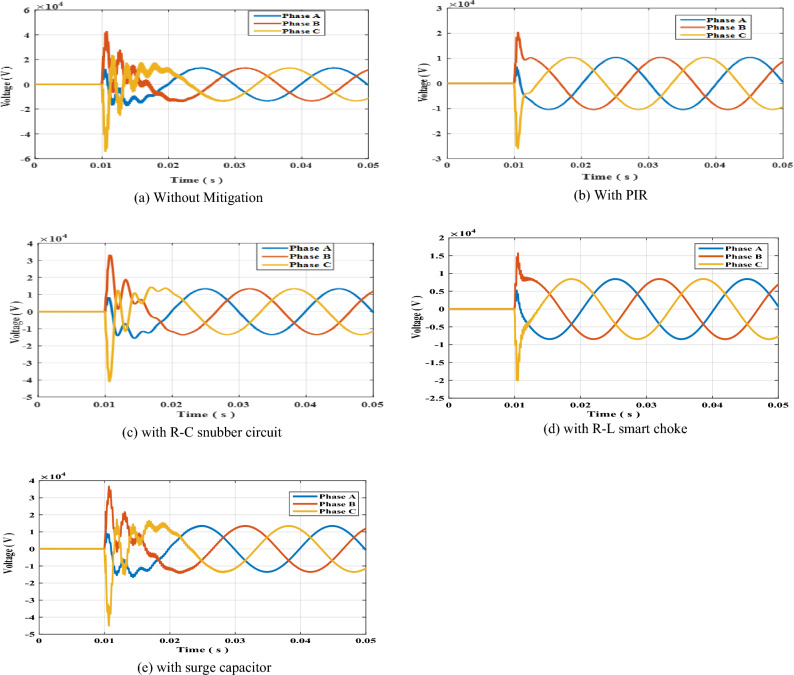
Switching overvoltage across circuit breaker S1 in radial topology.

The circuit breaker switch S1 shown in Fig. 1a for radial topology was suddenly opened at t = 10 ms. The results of the arising switching transient overvoltage is shown in Fig. 2a. The PIR was connected in parallel with S1 and effectively reduced the overvoltages as shown in Fig. 2b. Sequentially, the PIR was removed R–C snubber circuit was connected in parallel with resulting transient overvoltages shown in Fig. 2c. The same sequence was done for the smart choke and surge capacitor whose results are shown in Fig. 2d,e respectively. The peak values recorded for each phase in each mitigation scheme are presented in Table 3. The results show that R–L smart choke was the most effective scheme in reducing switching overvoltages. Further discussion and analysis of the results are presented in the next section. To Further elaborate the impact of R–L choke upon the resulting SOV, the values of R and L such that the value of R was increased to 100 Ω while keeping L constant at 1.5 mH, then the value of L was increased to 2 mH while keeping R constant at 50 Ω. The resulting SOV was recorded for each case. The results of SOVs with new R an L are presented in Table 4. The results show that the minimal SOV was for R = 50 Ω and L = 1.5 mH.

**Table 3:** Peak value of switching overvoltage across circuit breaker S1 in radial topology.

Mitigation method	Switching overvoltage (kV)		
	Phase A	Phase B	Phase C
Without mitigation	17.601	42.883	54.559
With PIR	6.631	20.628	26.120
With R–C	15.451	32.238	41.117
With choke coil R–L	5.394	15.867	20.236
With surge capacitor	16.675	36.756	45.332

**Table 4:** Peak value of switching overvoltage across circuit breaker S1 in radial topology with R–L choke connected.

R–L values	Switching overvoltage (kV)		
	Phase A	Phase B	Phase C
R = 50 Ω, L = 1.5 mH	5.394	15.867	20.236
R = 100 Ω, L = 1.5 mH	6.801	22.120	28.317
R = 50 Ω, L = 2 mH	5.756	16.709	21.411

### Simulation results for single-sided ring topology

**Figure 3:**
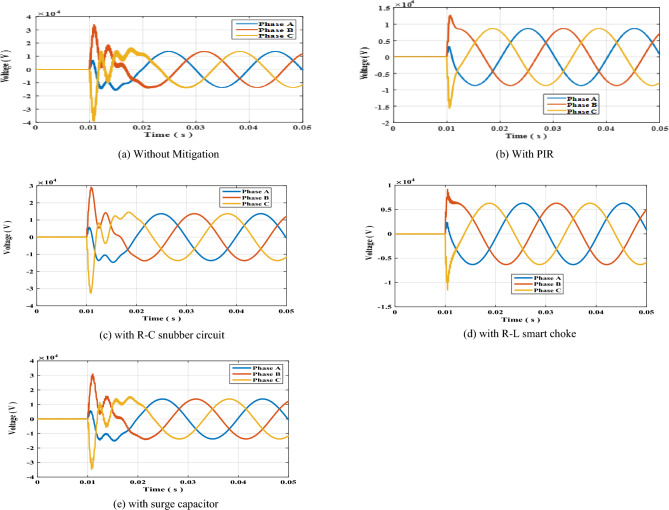
Switching overvoltage across circuit breaker S1 in single-sided ring topology.

The same sequence of the previous section will be undergone for this section for single sided ring topology such that circuit breaker switch S1 was opened at t = 10 ms. The results of the arising switching transient overvoltage without mitigation, with PIR, RC-snubber, smart choke and surge capacitor shown in Fig. 3a–e respectively. The peak values of the switching transients per phase are presented in Table 5. The results show that R–L smart choke was the most effective scheme in reducing switching overvoltages as in previous case. As in previous topology, the impact of R–L choke upon the resulting SOV was investigated by changing the values of R and L as presented in Table 6. The results show that the minimal SOV was for R = 50 Ω and L = 1.5 mH.

**Table 5:** Peak value of switching overvoltage across circuit breaker S1 in single sided ring topology.

Mitigation method	TRV (kV)		
	Phase A	Phase B	Phase C
Without mitigation	16.021	34.272	39.452
With PIR	3237	12,800	15,700
With R–C	14.700	29.216	32.775
With choke coil R–L	2.440	9.238	11.616
With surge capacitor	15.260	31.500	34.835

**Table 6:** Peak value of switching overvoltage across circuit breaker S1 in single sided ring topology with R–L choke connected.

R–L values	Switching overvoltage (kV)		
	Phase A	Phase B	Phase C
R = 50 Ω, L = 1.5 mH	2.440	9.238	11.616
R = 100 Ω, L = 1.5 mH	3.449	13.582	16.580
R = 50 Ω, L = 2 mH	2.613	9.707	12.237

### Simulation results for double-sided ring topology

**Figure 4:**
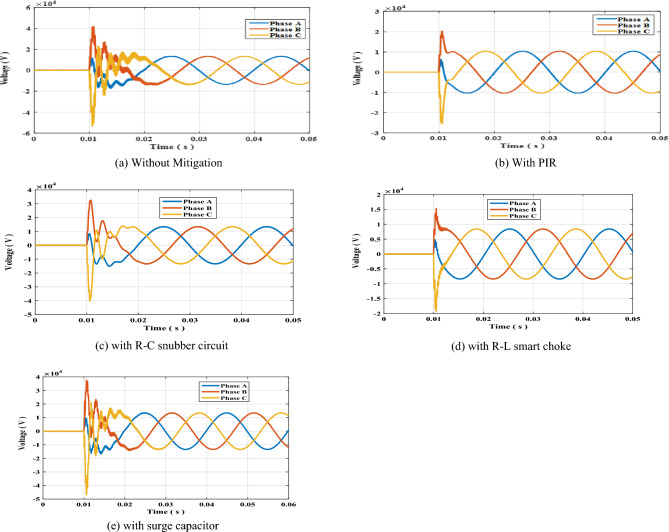
Switching overvoltage across circuit breaker S1 in double-sided ring topology.

The results of the arising switching transient overvoltage across S1 in double sided ring topology of Fig. 1c in case no mitigation used, with PIR, RC-snubber, smart choke and surge capacitor shown in Fig. 4a–e respectively. The peak values of the switching transients per phase are presented in Table 7. In the same manner as in previous topologies, the values of R and L were changed as presented in Table 8. The results show that the same conclusion as in previous topologies which is the minimal SOV was for R = 50 Ω and L = 1.5 mH.

**Table 7:** Peak value of switching overvoltage across circuit breaker S1 in double-sided ring topology.

Mitigation method	TRV (kV)		
	Phase A	Phase B	Phase C
Without mitigation	17.550	42.330	53.850
With PIR	6.418	20.460	25.736
With R–C	15.073	32.751	40.295
With choke coil R–L	4.885	15.452	19.551
With surge capacitor	16.844	37.832	47.237

**Table 8:** Peak value of switching overvoltage across circuit breaker S1 in double sided ring topology with R–L choke connected.

R–L values	Switching overvoltage (kV)		
	Phase A	Phase B	Phase C
R = 50 Ω, L = 1.5 mH	4.885	15.452	19.551
R = 100 Ω, L = 1.5 mH	6.418	21.660	27.507
R = 50 Ω, L = 2 mH	5.237	16.231	20.610

### Simulation results for star topology

**Figure 5:**
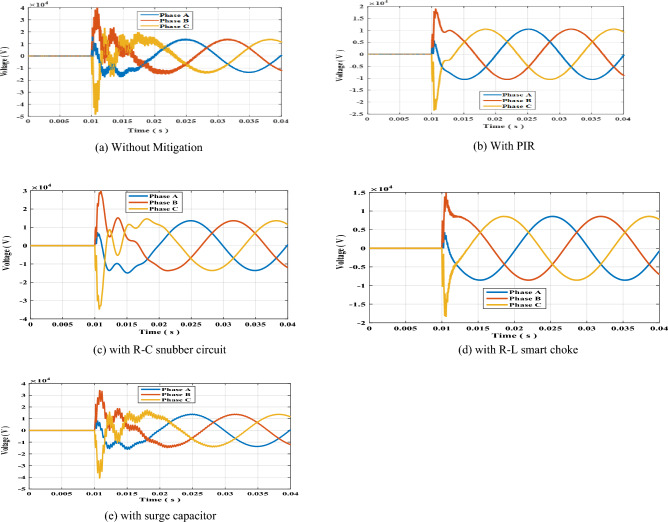
Switching overvoltage across circuit breaker S1 in star topology.

The results for the switching transient overvoltage across S1 in star topology of Fig. 1d in case no mitigation used, with PIR, RC-snubber, smart choke and surge capacitor shown in Fig. 5a–e respectively. The peak values of the switching transients per phase are presented in Table 9. In the same sequence for R and L testing was done for this topology as in previous topologies as presented in Table 10. The results show that the same conclusion as in previous topologies.

**Table 9:** Peak value of switching overvoltage across circuit breaker S1 in star topology.

Mitigation method	TRV (kV)		
	Phase A	Phase B	Phase C
Without mitigation	17.352	39.725	48.186
With PIR	5.583	18.952	23.394
With R–C	14.913	29.883	34.816
With choke coil R–L	4.309	14.921	18.439
With surge capacitor	16.135	34.132	40.941

**Table 10:** Peak value of switching overvoltage across circuit breaker S1 in star topology with R–L choke connected.

R–L values	Switching overvoltage (kV)		
	Phase A	Phase B	Phase C
R = 50 Ω, L = 1.5 mH	4.309	14.921	18.439
R = 100 Ω, L = 1.5 mH	5.967	20.000	24.741
R = 50 Ω, L = 2 mH	4.543	15.744	19.544

## Discussion and effectivity analysis

**Figure 6:**
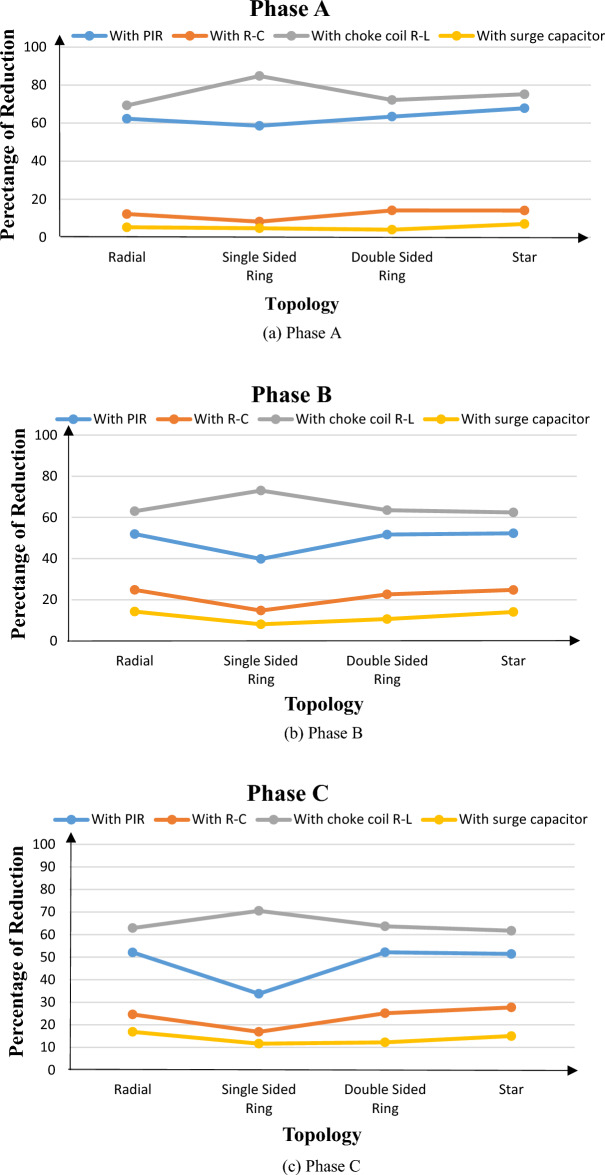
Percentage of reduction in switching overvoltages due to different mitigation schemes in each topology.

To evaluate the effectivity of each mitigation scheme used, the amount of reduction in switching overvoltages was computed. For radial topology, the transient overvoltage of phase A reached a peak value of 17.6 kV without any mitigation technique used. That value was reduced to 6.6 kV for the same phase when PIR was used. Hence, the percentage of the reduction in the switching overvoltage in this case is 62.32% with respect to the unmitigated original overvoltage of 17.6 kV. The percentage of reduction for phase A for each mitigation scheme and in each topology are shown in Fig. 6a. Similarly, for phases B and C in Fig. 6b,c respectively. The results show that smart choke R–L was the most effective scheme for all topologies. That effectiveness was monitored from the percentage of overvoltage that reached 84.7% in case of single sided ring topology and ranged from 62 to 73% in other topologies. The remaining schemes could be arranged in order of their effectivity as PIR, R–C snubber and surge capacitor respectively. The PIR, R–C snubber and surge capacitor had a percentage reduction ranging from 33 to 67%, 8 to 25% and 4 to 15% respectively. It could be noticed also, that the order of effectiveness of mitigation schemes remained the same for all topologies.

**Figure 7:**
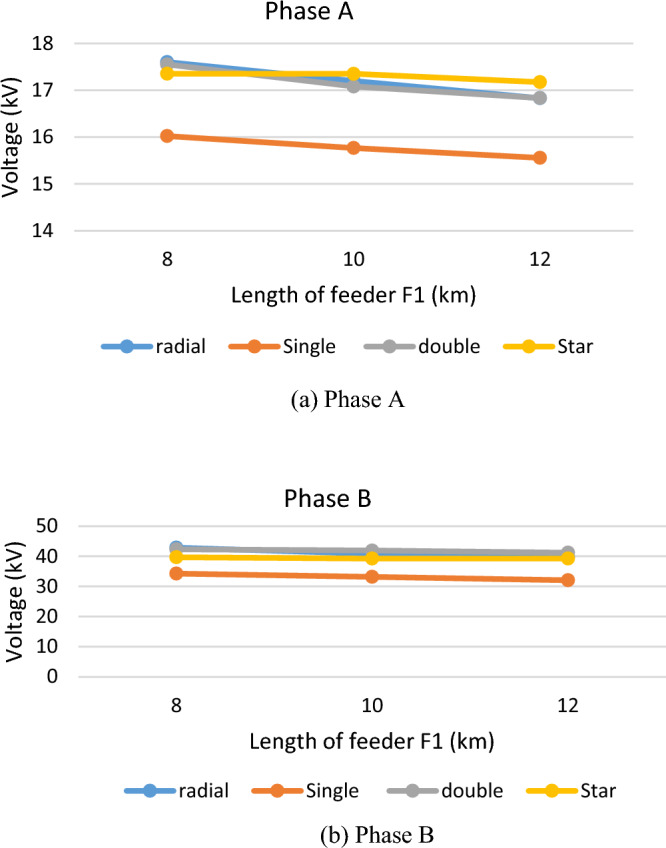
The transient overvoltage in each topology for different lengths of feeder F1.

The effect of increasing the feeder length was also investigated by increasing the length of feeder F1 from 8 to 10 km and 12 km. The increase in feeder length did reduce the switching overvoltage but that reduction was very limited as shown in Fig. 7. The figure shows the level of switching overvoltage for each topology for feeder F1 of 8 km, 10 km and 12 km. it could be seen from the figure that the impact of the feeder length was very limited.

## Conclusions

The expanding utilization of wind farms and their various topologies has mandated the study of the effectiveness of the different overvoltage suppression schemes within each topology. In this study, four different topologies were selected, radial, single sided ring, double sided ring and star topologies. For each topology, the simulation tests showed a significant transient overvoltage showing during the switching of the circuit breaker. To suppress these overvoltages, four different mitigation schemes including pre-insertion resistor (PIR), RC-snubber circuit, R–L choke coil and surge capacitor.

To evaluate the effectiveness of each mitigation scheme, the percentage of reduction in transient overvoltage was computed in each case. The percentage of reduction in switching overvoltage showed the R–L choke coil to be the most effective mitigation scheme with percentage of reduction ranging from 62 to 84%. The PIR, R–C and snubber had a percentage reduction ranging from 33 to 67%, 8 to 25% and 4 to 15% respectively. It could be concluded that the effectiveness of the schemes could be arranged in order of their effectiveness as R–L choke coil, PIR, R–C snubber and surge capacitor respectively. Additional investigation showed the value of R for the choke coil to be highly effective at 50 Ω. The inductance of the choke coil showed high effectiveness at 1.5 mH. The previous R–L values showed a 62–84% which were the highest percentage of reduction among all mitigation measures. Also, it was noticed that changing the wind farm topology did not affect the previous order of effectiveness of mitigation schemes.

## Data Availability

The data that support the findings of this study are available from the corresponding author upon reasonable request.

## Abbreviations

CB: Circuit breaker
DSR: Double sided-ring
${\mathrm{g}}_{\mathrm{m}}$: g_m_ Conductance of the arc
${\mathrm{P}}_{\mathrm{H}}$: P_H_ Heating power of arc
$${\mathrm{P}}_{\mathrm{o}}$$: P_o_ Cooling power
PIR: Pre-insertion resistor
Q: Energy stored into the arc
$\uptau$: τ Arc time constant
SSR: Single sided-ring
